# Rare diseases and orphan drugs: Latvian story

**DOI:** 10.1186/s13023-014-0147-z

**Published:** 2014-09-18

**Authors:** Konstantins Logviss, Dainis Krievins, Santa Purvina

**Affiliations:** Department of Internal Diseases, Division of Pharmacology, Riga Stradins University, 13 Pilsonu Street, Riga, LV-1002 Latvia; Department of Education and Science, Pauls Stradins Clinical University Hospital, 13 Pilsonu Street, Riga, LV-1002 Latvia; University of Latvia, 19 Raina Boulevard, Riga, LV-1586 Latvia

**Keywords:** Rare diseases, Orphan drugs, Availability, Accessibility, Latvia

## Abstract

**Background:**

Ten years have passed since Latvia became a Member State of the EU in 2004. As a result European regulations, including those related to rare diseases and orphan drugs, have been applied to Latvian legislative system. Orphan diseases have been recognized as a priority area for action in the public health system, though there are significant differences in the national healthcare services for rare diseases among the EU States. This study aims to determine situation in the field of rare diseases in Latvia and compare it with other European countries.

**Methods:**

We used the national plan for rare diseases, EUCERD reports, Orphanet data, Latvian and European regulations, publicly available data from the state agencies, and directly contacted drug manufacturers and wholesalers.

**Results:**

National plan for rare diseases was developed and approved in 2013. Although there are no official designated centers of expertise as well as no specific register for rare diseases. Newborns are screened for only two disorders: phenylketonuria and congenital hypothyroidism. Currently 34 orphan drugs are available on Latvian market. Three medicines (8.8%) are included in the reimbursement drug list, all indicated for Ph + CML. 15 drugs (44.1%) were reimbursed within the framework of individual reimbursement system, and five drugs (14.7%) were provided within the program of medicinal treatment of rare diseases in children.

**Conclusions:**

Majority of orphan drugs authorized in the EU are not available in Latvia, moreover those drugs that are available are often not accessible because they are insufficiently reimbursed. Besides, approval of the national plan might be an important step towards improving situation in the field of rare diseases.

## Background

Ten years have passed since Latvia became a member of the European Union (EU) in 2004. Since then the EU laws and regulations, including those related to rare diseases, have been applied to Latvian legislative system. According to the EU regulations disease is considered as rare if it affects not more than five in ten thousand people [1]. Most patients suffer from even rarer disorders affecting one person in 100 000 or more [2], and it could be very difficult to diagnose and manage these conditions in relatively small populations. It is estimated that between five and eight thousand different rare conditions exist, affecting 6-8% of the population, concluding that about 30 million people are suffering from rare disorders in the EU. Rare diseases are life-threatening or chronically debilitating conditions of different origin. The majority of them are genetic disorders, others being rare cancers, autoimmune, toxic and infectious diseases [3].

The EU offers a number of incentives to promote the development of orphan medicines since, under normal market conditions, pharmaceutical companies have little interest in developing products intended for small numbers of patients. The incentives include assistance in drug development, reduced fees for marketing authorization, and protection from market competition once the drug is authorized (10 years of marketing exclusivity) [1]. Orphan designation refers to awarding of orphan status to a drug, but marketing authorization refers to the approval to market the product. Applications for orphan designation are examined by the European Medicines Agency’s (EMA) Committee for Orphan Medicinal Products (COMP), which adopts an opinion that is forwarded to the European Commission, which afterwards decides whether to grant an orphan designation for the drug in question. Then pharmaceutical company submits a single marketing authorization application to the EMA under the centralized procedure. Once granted by the European Commission, a centralized marketing authorization for orphan medicinal product is valid in all the EU states. While many medicines may have received an orphan designation, few have received a marketing authorization. As of April 2014 there were more than a thousand positive opinions on orphan designation and only 72 orphan drugs authorized in the EU [4,5].

Generally there is a limited public awareness of the rare diseases. The national healthcare services for these disorders differ significantly among the EU countries, resulting in unequal access to diagnostics and treatment (including orphan medicines). An orphan drug is generally considered to be available when it is market authorized and priced. To be accessible, however, it needs to be reimbursed by public fund. Considering all the above mentioned European Council recommended Member States to establish and implement national plans for rare diseases by the end of 2013 [2].

### Aim of the study

This study aims to determine situation in the field of rare diseases in Latvia and compare it with other European countries. It includes visibility of these diseases, public awareness, national strategy, research, diagnostics and treatment, including availability and accessibility of orphan drugs.

## Methods

The following sources of information were used to evaluate situation in the field of rare diseases in Latvia: National Plan for Rare Diseases (http://www.vm.gov.lv), EUCERD 2012, 2013 and 2014 Reports on the State of the Art of Rare Disease Activities in Europe (http://www.eucerd.eu), Statistics of the Center for Disease Prevention and Control (http://www.spkc.gov.lv), Orphanet Latvia (http://www.orpha.net/national/LV-LV) and laws and regulations of the Cabinet of Ministers (http://likumi.lv). A literature review was performed to compare Latvian situation in the field of orphan medicines with other European countries.

Orphan drugs authorized in the EU were identified by using EMA’s list of rare disease designations (http://www.ema.europa.eu) and European Community Register of designated orphan medicinal products (http://ec.europa.eu/health). Availability of orphan drugs on Latvian market was determined by using the National Register of Human Medicines maintained by the State Agency of Medicines of Latvia (http://www.zva.gov.lv), as well as EUCERD 2012, 2013 and 2014 Reports (http://www.eucerd.eu), and directly contacting drug manufacturers and wholesalers. Drugs were considered to be available if they were marketed/launched in Latvia according to data in at least two of the above mentioned sources. The National Health Service (http://www.vmnvd.gov.lv) data were used to assess drug reimbursement including the national reimbursement list (as of January 2014) and individual reimbursement data in 2008-2012. Children’s Clinical University Hospital purchase procedure reports on “Medicinal treatment for children with rare diseases” program in 2010-2014 (http://www.bkus.lv) were analyzed to identify orphan drugs provided within this program. Drugs were considered to be accessible if they were provided within some of the reimbursement mechanisms mentioned above.

## Results

### National plan for rare diseases

Latvian national plan for rare diseases was written by the working group, which included health care professionals, representatives from the Ministry of Health (MoH) and patient organizations. The plan was submitted to the MoH for evaluation in December 2011 [6], and a public consultation of the plan was launched in 2012 and the results were subsequently analyzed by the MoH. A number of meetings with different stakeholders were held, and as a result, the MoH developed and on 20 June 2013 approved the National Plan for Rare Diseases for the period from 2013 to 2015 [7] in accordance with the European Council recommendations of 8 June 2009 on an action in the field of rare diseases [2].

There is no official definition for rare diseases in Latvia, as stakeholders accept definition of the European Regulation on Orphan Medicinal Products of a prevalence of no more than 5 in 10 000 individuals, and that rare diseases are life-threatening or chronically debilitating [6,7]. Main strategic objectives of the plan are related to improving access to information on rare diseases for health care professionals and patients with their families, as well as creation of rare disease patient register. An important role of the plan is dedicated to the prevention and early detection of rare diseases, integrated medical and social care for patients, and continuing education of health care professionals in the field of rare diseases.

### Centers of expertise

There are currently no official designated centers of expertise for rare diseases in Latvia, but a meeting was held in 2013 to discuss possible criteria for national centers of expertise [8]. A legal framework for centers of expertise, including those for rare diseases, is expected in the future. Several medical centers, that fulfill this role, are currently recognized by reputation only [6]. For example, the Children’s Clinical University Hospital (CCUH) provides genetic services, as well as services for children with haematological, oncological and endocrinological diseases. Riga East Clinical University Hospital (RECUH) has a specialized clinic of chemotherapy and haematology, in which patients with haemophilia A and B, factor XII deficiency and von Willebrand disease receive diagnostics and treatment. Rare oncological diseases, e.g. Burkitt’s lymphoma, Langerhans cell histiocytosis, Mantle-cell non-Hodgkin’s lymphoma, multiple endocrine neoplasia, Ewing’s sarcoma, Wilms’ tumour, Waldenstrom’s macroglobulinaemia and others can be treated in this hospital as well. Pauls Stradins Clinical University Hospital (PSCUH) provides services in different rare disease areas, such as cardiology, angiology, pulmonology, nephrology, endocrinology, gastroenterology, oncology and ophthalmology. Latvian Pulmonary Hypertension Center is a part of Center of Cardiology of Latvian University located in PSCUH. The MoH, Orphanet Latvian team and experts from the three university hospitals mentioned above met in 2013 and started work on developing criteria for national centers of expertise [8].

### Patient registers

Currently there is no specific register for rare disease patients in Latvia making it impossible to fully collect and evaluate information on rare diseases, although the national plan for rare diseases foresees activities for evaluation and improvement of existing patient registers to start centralized data collection on rare disease patients [7,8]. Register of Patients Suffering from Certain Diseases maintained by the Center for Disease Prevention and Control (CDPC) contains records on some rare diseases including cancers and congenital anomalies. Congenital Anomaly Register is a part of Register of Patients Suffering from Certain Diseases and it contained data on 11 990 patients with congenital anomalies as of June 2012 [9]. According to data from the Newborn Register 582 cases of congenital malformations were diagnosed in maternity wards in 2011 and 677 cases in 2012 [10]. They calculated in 6.1% of total newborns morbidity rate in 2012. However, data in the Congenital Anomaly Register is incomplete, since the register is not functioning optimally, because there is no mechanism in place that would regulate flow of information on newly diagnosed congenital anomalies [7]. Therefore the register in its current form requires big improvements as it is stated in the national plan for rare diseases. Genome Database of Latvian Population has been created and is maintained by Latvian Biomedical Research and Study Center (LBRSC) [11]. This database is a nationwide project designed to create identification, storage and processing system for health and genetic information of Latvian population for research, preventive and therapeutic purposes. As of November 2012 the number of participants recruited in the project (number of collected DNA samples) was 20 126 [12]. DNA and data collection of monogenic diseases maintained by Genome Database of Latvian Population contains more than 800 patient samples (more than 500 of which for rare diseases) [7].

Rare cancers are included in the National Cancer Control Program (2009-2015) adopted by the Cabinet of Ministers in 2009 [13]. According to data of Latvian Cancer Register there were 71 166 patients registered at the end of 2012 [14] (cancer was newly diagnosed in 11 534 patients in 2012). Taking into account that rare cancers calculate in 24% of the total cancer prevalence in the EU [15] (annual incidence rate of rare cancers in Europe is 22% of all cancer diagnoses), the estimated number of patients with rare cancers in Latvia could be around 17 080 patients (annually around 2 537 newly diagnosed patients).

The Cardiovascular Health Improvement Action Plan (2013-2015) was adopted in 2013 [16]. It includes activities in the fields of health promotion, improving cardiovascular disease treatment and early diagnostics of congenital malformation of the heart. According to data from the heart disease register of the CCUH clinic of children’s cardiology and cardiac surgery 152 newborns (0.77%) were diagnosed with congenital heart disease in 2012, 11.1% of heart pathologies were diagnosed late and 55.5% of pathologies were diagnosed antenatally.

Health care professionals from several university hospital centers collect rare disease patient data, for example, since 2007 there is a Pulmonary Arterial Hypertension (PAH) patient register at the Latvian Cardiology Center of PSCUH [6]. Center of endocrinology of PSCUH has created several data bases of patients with rare endocrine diseases, such as acromegaly, Cushing’s disease, and MEN (multiple endocrine neoplasia) syndrome [8]. These data bases were created for follow-up purposes, as well as to serve as a source of scientific information. Congenital anomaly register is held by the medical genetics clinic at the CCUH. According to CCUH data there are records on 40 cystic fibrosis patients in Latvia as of the end of 2013 [17], and every year this diagnosis is confirmed in one child, on average. Data are also collected by rare disease patient organizations. For instance, according to Latvian Haemophilia Society data there are around 250 patients with bleeding disorders [7], while Pulmonary Hypertension Association has data on 51 patients with diagnosed PAH.

It should be noted that implementation of the e-health project is planned to be launched in Latvia in 2014. Within this project data on patient health and received health care services will be stored centrally. There is also a pilot plan to use Orpha codes and OMIM codes for rare diseases within the e-health system to ensure identification and visibility of rare diseases [8]. Some activities are currently being implemented including the approval of an act concerning the plan to include Orpha codes in the congenital anomaly and cancer registers. It would allow using e-health system information for obtaining data on prevalence, diagnostics, and treatment of rare diseases. If the above mentioned plan was implemented, making new separate rare disease patient register would not necessarily be required [7].

Latvia contributed to EUROCARE-5 (European cancer register based study on survival and care of cancer patients), RARECARENet (information network on rare cancers), EUROCAT (European surveillance of congenital anomalies), and EUHASS (European haemophilia safety surveillance) [6]. Latvian teams also participated in the following pilot European reference networks for rare diseases: Dyscerne (European network of centers of expertise for dysmorphology) and PAAIR (patient associations and alpha-1 international register) [18]. These pilot projects were financed by the European Commission within the scope of the Community action program on rare diseases, including genetic diseases (1999-2007) and the second program of the Community action in the field of health (2008-2013). Only one register of rare disease patients (Latvian cystic fibrosis patient register) is listed in Orphanet report on rare disease registers in Europe [19], which means that there is a need to improve information exchange and optimize flow of information among different institutions.

### Diagnostics

Newborns are screened for only two rare disorders in Latvia: phenylketonuria (since 1987) and congenital hypothyroidism (since 1996) [7]. Data on newborns screened in maternity units are collected in the newborns register that is supervised by the CDPC [8]. An average of 22 neonates with congenital hypothyroidism and 4 with phenylketonuria were annually diagnosed through this neonatal screening program in 2007-2012 [20]. A question has been recently raised concerning expanding the newborn screening by using tandem mass spectrometry method [7]. Latvian Food and Veterinary Service and Institute of Organic Synthesis have such mass spectrometry devices, but they are not used for medical purposes making it a nonsense situation.

Some prenatal and postnatal diagnostic tests for rare genetic diseases are financed by the National Health Service (NHS) [7], including:

1. Cytogenetic analysis: chromosome analysis with the standard method and molecular cytogenetic or FISH (fluorescence in situ hybridization) method;
2. Genetic biochemical analysis: biochemical screening of pregnant women at high risk for fetal genetic abnormalities; neonatal screening for phenylketonuria and congenital hypothyroidism; selective screening of inborn metabolic disorders (spectrum of amino acids and organic acids, as well as analysis of oligosaccharides, mucopolysaccharides and carbohydrates);
3. DNA diagnostics for: spinal muscular atrophy, hereditary motor and sensory polyneuropathy, long chain and medium chain fatty acid oxidation disorders, Huntington’s chorea, fragile X syndrome.

Additionally, diagnostics of the following genetic disorders are available within the scientific research projects or in laboratories of scientific institutions [7]: cystic fibrosis, hereditary haemochromatosis, Wilson’s disease, Gilbert’s syndrome, alpha-1 antitrypsin deficiency, several forms of inherited cancers, and thrombophilia. In some cases clinical university hospitals collaborate and send samples to LBRSC for genetic testing [8]. For example, genetic testing of all family members of patients with MEN syndrome is financed in terms of scientific project. Genetic testing is available through Medical Genetics Clinic of CCUH, Molecular Genetics Scientific Laboratory of Riga Stradins University (RSU), and LBRSC [6]. In recent years amount of newly diagnosed genetic diseases has increased by about 25% [7] thanks to new diagnostic methods offered by these institutions. However, despite the progress made in the diagnostics of genetic diseases, number of patients with unspecified genetic abnormalities requiring additional investigation abroad is increasing. Genetic testing in other EU states is possible through the special E112/S2 forms, if genetic testing is a health care service usually financed from the state budget, but this service cannot be provided in Latvia or cannot be provided within a reasonable period of time [6]. Mostly it is provided for children with life-threatening or treatable conditions.

Clinical guidelines for rare diseases have not been approved at the national level, although center of endocrinology of PSCUH in collaboration with endocrinologists from RECUH and CCUH issued diagnostic algorithms for rare endocrine diseases in 2013 [8]. These recommendations aim to help general practitioners and endocrinologists to consider rare endocrine diseases in certain types of patients. PSCUH also organizes post-diploma educational courses in most areas of medicine, including endocrinology. These courses usually cover not only common clinical conditions but rare diseases as well. As it is with some rehabilitation services described below, even in such small country as Latvia there are some regional differences in availability of diagnostics for rare diseases [7], because Latvian health care system is organized in such a way that individual tests requiring modern technologies and complex manipulations are performed only by the university hospitals, which are concentrated in Riga. Further medical care with less complex health services is provided by regional multi-profile hospitals.

### Rehabilitation services

In parallel with timely and accurate diagnostics and appropriate treatment, rare disease patients require also proper rehabilitation services. State funding of medical rehabilitation and amount of provided services, including funding for assistive technologies (technical aids), has decreased significantly over the past few years [7]. Therefore current availability of rehabilitation services for rare disease patients is not sufficient. For instance, suitable solutions are still not found for such assistive technologies as special mobility devices for muscular dystrophy patients and oxygen devices for pulmonary hypertension patients. This problem is particularly actual outside Riga, where rehabilitation services are limited. Palliative care services for children are provided by a multidisciplinary palliative care team set up in CCUH. This team provides inpatient and outpatient consultative services to families as well as palliative care services at home for children in Riga. Palliative care services for children in other Latvian regions and cities (except Liepaja) are limited, despite the fact that the government supports development of these services. The reason of such differences could be partially explained by the fact, that there are certain requirements for the health care specialists to provide these services, as well difficulties are experienced in involvement of multidisciplinary team.

A new service for persons with disabilities (including disabilities due to rare diseases) was launched in January 2013 [8]. It is a municipality based service providing an assistant for performing activities outside home, e.g. to get to work, school or rehabilitation institution. The assistant service is eligible for persons from 18 years of age with group 1 or group 2 disability (very severe or severe disability, respectively), or persons aged 5-18 years without dividing disabilities into the groups. The assistant service is provided on the basis of conclusion of the state medical commission for the assessment of health condition and working ability.

### Patient organizations

Latvian rare disease patient organization “Caladrius” was launched in 2009 [6,18]. Mission of this organization is to provide patients with relevant information in the field of rare diseases, as well as to support patients and represent their interests. “Caladrius” established a fund to help rare disease patients who could not otherwise pay for their treatment, and in collaboration with CCUH organized two visits of highly qualified cardiac surgeons in 2011. As a result complicated operations were carried out for eleven children with inborn heart pathologies.

There is a number of other rare disease related patient organizations in Latvia, including Haemophilia Society, Pulmonary Hypertension Association, Cystic Fibrosis Society, and Association of People with Special Needs “Motus Vita” [8]. These organizations often collaborate with each other, organize rare disease events and celebrate annual rare disease day. For example, Pulmonary Hypertension Association financially supported the first pulmonary endarterectomy for chronic thromboembolic pulmonary hypertension (CTEPH) patient in 2013, organized the summer health camp, and proceeded the oxygen home care therapy supporting for PAH patients. Latvian Haemophilia Society intensified cooperation with Lithuanian Haemophilia Society in 2013 in order to provide disease specific training for physiotherapists working with people with bleeding disorders in CCUH and RECUH. There are some plans to create an alliance of rare disease patient organizations and chronic patient organizations at national level. Until now, there were 8-9 organizations that share information and collaborate together in this area.

### Sources of information on rare diseases

MoH has designated the CDPC as a representative of Latvia to participate in Orphanet Europe Joint Action project [6]. Orphanet team is currently hosted by the CDPC and is in charge of collecting data on rare disease related services in Latvia, such as specialized clinics, medical laboratories, ongoing research, registers, clinical trials and patient organizations. Orphanet national website was launched in April 2012 and is regularly updated by the Orphanet team. Web based information is also available for a limited number of conditions through other sources of information on rare diseases, such as paediatric rheumatic diseases, lysosomal storage diseases, and through websites of patient organizations for pulmonary hypertension, cystic fibrosis, neuromuscular diseases, bleeding disorders, leukaemia and some other forms of cancer.

### Research

Generally funding is available for rare disease projects (through state budget, charities and pharmaceutical companies) [6] although these funds are not specifically designed for rare disease research. Rare diseases were not included in the priority directions of science and research [8], but a number of scientific research projects in the field of rare diseases take place in Latvia [7]. As well collaboration networks have been created with researchers from Baltic States and other countries around the world. Examples of such projects are researches in:

1. Hereditary liver, lung and mitochondrial diseases (Scientific Laboratory of Molecular Genetics, RSU);
2. Genetics of inherited cancers (Institute of Oncology, RSU);
3. Nonsyndromic hearing loss (Clinic of Medical Genetics, CCUH);
4. Nonsyndromic orofacial clefts, familial melanoma, MODY (maturity onset diabetes of the young) type diabetes, congenital muscular dystrophies and familial hypercholesterolaemia (LBRSC);
5. Genetics and effectiveness of treatment of acromegaly (PSCUH and LBRSC);
6. Genome analysis of rare hereditary eye disorders and metabolic abnormalities (collaborative project with the University of Tartu).
7. Rare cardiovascular diseases. Project coordinated by Center for Rare Cardiovascular Diseases, John Paul II Hospital in Krakow, Poland. Principal partners are Latvian Center of Cardiology, PSCUH and Lithuanian University of Medical Sciences, Kaunas.

Baltic Metabolic Group annually brings together experts in the field of metabolic diseases [7]. Research projects on inherited metabolic diseases in the Baltic region are carried out within the framework of this group. Latvia is currently an observer of the E-Rare (ERA-Net for research programs on rare diseases) project [8], however Latvian funding agencies do not contribute to the IRDiRC (International Rare Disease Research Consortium) project.

### Orphan medicinal products

Pharmaceutical companies submit a single marketing authorization application to the EMA under the centralized procedure. Once granted by the European Commission, a centralized marketing authorization for orphan drug is valid in all the EU states (including Latvia). Whereas decisions surrounding orphan designation and marketing authorization of orphan medicines are taken at the EU level, decisions governing pricing and reimbursement of orphan drugs are a member state responsibility. State Agency of Medicines (SAM) of Latvia includes medicinal products registered in Latvia as well as medicines centrally registered by the EMA in a register of medical products of the Republic of Latvia [6]. SAM is also responsible for regular collection and distribution of information on drugs, including orphan medicinal products, as well as for compiling information on drug safety, evaluating possible risks and coordinating measures for risk reduction. Inventory of Community and Member States’ incentive measures to aid the research, marketing, development and availability of orphan medicinal products reported that in Latvia SAM is entitled to make a decision regarding the fee exemption or reduction for activities associated with the evaluation or registration of a medicinal product if it (with or without orphan designation) is intended to the treatment of a rare disease [21]. The Inventory also stated that SAM may issue distribution authorization for medicinal product not registered in Latvia if it is intended for treatment of a rare disease (for an individual patient on the basis of prescription or for use in a health care institution on the basis of a written request). A first level of accessibility exists for orphan medicines that have not yet been authorized, the most common being compassionate use. It covers diseases for which no satisfactory alternative therapy exists. SAM has approved several programs for drug compassionate use in Latvian hospitals [22]. The programs include influenza drugs (oseltamivir and zanamivir) for intravenous administration, medicine for chronic hepatitis C (boceprevir), and drugs for cancer (Sprycel and Votrient) used in Ph + CML and metastatic soft tissue sarcoma, respectively. Sprycel is an orphan drug included in the national reimbursement list C, while Votrient was originally designated an orphan medicine, but it was further withdrawn from the EU register of designated orphan medicinal products upon request of the sponsor.

### Orphan drug availability

Currently 34 orphan drugs are available on Latvian market (Table 1), including 6 drugs that are no longer considered to be orphan medicines in the EU. These drugs were originally designated orphan medicines, but further withdrawn from the EU register of designated orphan medicinal products either upon request of the sponsor (Afinitor, Glivec, Revolade and Sutent) or at the end of the period of market exclusivity (Aldurazyme and Ventavis). Strictly speaking it means that only 28 pure orphan products are available in Latvia calculating in 38.9% out of 72 orphan drugs currently authorized in the EU. Orphan medicines are distributed by both hospital and community pharmacies. It should be noted that some of the orphan drugs were stated as available in SAM medicinal product register, however they were not considered to be available in Latvia as they did not meet availability criteria defined in current study (i.e. drugs were considered to be available if they were marketed/launched in Latvia according to data in at least two of the sources mentioned in the Methods section). For example, Torisel is stated as available in SAM register, although drug manufacturer has confirmed the opposite. Additionally individual reimbursement data show that Torisel was reimbursed for two patients with malignant neoplasm of kidney in 2008. According to the company, Somavert (which is no longer an orphan medicine in Europe due to expiration of the period of market exclusivity) was available shortly after registration, but there was no demand, so the product currently is not available. As stated by the wholesalers, Tracleer and Vidaza were once in stock but are not available anymore. The manufacturers also confirmed that these products are not available in Latvia.

**Table 1:** **Orphan drugs available in Latvia**

**Trade name**	**Active substance**	**Indication**	**Reimbursement**
Afinitor*	Everolimus	Neuroendocrine tumours of pancreatic origin (pNET)	
		Renal cell carcinoma (RCC)	
Aldurazyme*	Laronidase	Mucopolysaccharidosis I (MPS I)	CCUH
Arzerra	Ofatumumab	CLL	Individual
Atriance	Nelarabine	T-cell ALL	Individual
		T-cell lymphoblastic lymphoma	
Carbaglu	Carglumic acid	Hyperammonaemia	
Cystadane	Betaine anhydrous	Homocystinuria	CCUH/Individual
Diacomit	Stiripentol	Severe myoclonic epilepsy in infancy (Dravet’s syndrome)	Individual
Elaprase	Idursulfase	MPS II (Hunter syndrome)	CCUH
Evoltra	Clofarabine	ALL	
Exjade	Deferasirox	Beta thalassaemia major with iron overload due to blood transfusion	Individual
Gliolan	5-aminolevulinic acid hydrochloride	Malignant glioma	
Glivec*	Imatinib	Ph + CML	List A
		GIST	(previously list C)
		Dermatofibrosarcoma protuberans	Individual
		Ph + ALL	
		Hypereosinophilic syndrome	
		Myelodysplastic/ myeloproliferative diseases	
Increlex	Mecasermin	Primary insulin-like growth factor 1 deficiency	CCUH
Jakavi	Ruxolitinib	Myelofibrosis	
Kuvan	Sapropterin	Phenylketonuria	CCUH
		Tetrahydrobiopterin deficiency	
Litak	Cladribine	Hairy cell leukaemia	
Mozobil	Plerixafor	Haematopoietic stem cell transplantation in lymphoma or multiple myeloma patients	Individual
Myozyme	Alglucosidase alpha	Pompe disease	
Nexavar	Sorafenib	Hepatocellular carcinoma	Individual
		RCC	
Nplate	Romiplostim	ITP	Individual
Orfadin	Nitisinone	Hereditary tyrosinaemia type 1	
Pedea	Ibuprofen	Patent ductus arteriosus in preterm newborns	
Peyona	Caffeine citrate	Primary apnea of premature newborns	
Revatio	Sildenafil	PAH	Individual
Revolade*	Eltrombopag	ITP	Individual
Sprycel	Dasatinib	Ph + CML	List C
		Ph + ALL	Individual
Sutent*	Sunitinib	GIST	Individual
		RCC	
		pNET	
Tasigna	Nilotinib	Ph + CML	List C
Tobi Podhaler	Tobramycin	Pseudomonas aeruginosa infection in cystic fibrosis patients	
Ventavis*	Iloprost	PAH	
Volibris	Ambrisentan	PAH	Individual
Votubia	Everolimus	Renal angiomyolipoma or subependymal giant cell astrocytoma associated with tuberous sclerosis complex	
Wilzin	Zinc acetate dihydrate	Wilson’s disease	Individual
Yondelis	Trabectedin	Soft tissue sarcoma	
		Ovarian cancer	

*Afinitor, Aldurazyme, Glivec, Revolade, Sutent and Ventavis are no longer considered to be orphan medicines in the EU.

There is no information on Gliolan availability provided in the SAM register, although the product is stated as available in EUCERD reports, and availability was also confirmed by the manufacturer and the wholesaler. There are special requirements for use of Gliolan. It should only be used by experienced neurosurgeons who have completed a training course in fluorescence-guided surgery (fluorescence microscope is used in the procedure) in malignant glioma resection. The Marketing Authorization Holder (MAH) is obligated to implement mentioned training course. According to information provided by the MAH there is one neurosurgeon in Latvia experienced in utilizing the product. The SAM register provides no information on availability of Diacomit, however the product is stated as available in EUCERD reports, as well it was individually reimbursed annually in 2008-2012. There is also no information on Aldurazyme availability in the SAM register, although the company confirmed that the product is available. Aldurazyme is also stated as available in the EUCERD 2013 report. Additionally, it was purchased by CCUH within the “Medicinal treatment for children with rare diseases” program in 2014.

For some orphan drugs the manufacturers stated that these products would be available on demand if there was a patient requiring them, as it is in case of Atriance, Orfadin, Yondelis, Savene, Litak and Vpriv. Yondelis has been provided by the company free of charge for one time, but it did not affect further availability of the drug. Several packages of Ventavis are bought quite rarely and are available only for patients in PSCUH. Afinitor is also available, and according to the company’s information there could be around six patients in Latvia requiring treatment with this drug.

### Orphan drug pricing and reimbursement

There is no specific policy in place for pricing of orphan drugs in Latvia. Costs related to rare diseases and orphan drugs are currently included in the national health care budget [6,7]. Drug reimbursement covers drugs which are included in the national reimbursement drug list, or based on the medical council’s decision, drugs can also be reimbursed within the framework of individual reimbursement system with a limit of 14 229 Euro per patient per year [23]. The main principle of drug inclusion in the reimbursement list is that the drug should be therapeutically and cost effective, i.e. decision is value based and is not specific to orphan drugs. The national reimbursement list consists of three parts: list A covering therapeutically equivalent drugs (generics); list B that consists of drugs without therapeutic equivalent; and list C that contains drugs for which the annual cost exceeds 4 269 Euro per patient and the manufacturer is obliged to cover treatment expenses for a certain number of patients with his own resources (not less than 10%). The NHS evaluates therapeutic value, price, expected budget impact and cost-effectiveness for each drug before it is included in the reimbursement list. Drug price is compared with the prices in other EU countries. The price of the reimbursed medicine should not be higher than the third lowest price in the Czech Republic, Denmark, Romania, Slovakia and Hungary, and shall not exceed the price of the medicine in Estonia and Lithuania.

Health care of rare disease patients under 18 years of age is currently evaluated satisfactorily [7]. Since 2009, several orphan medicinal products for children are provided as a part of the state funded program “Medicinal treatment for children with rare diseases” coordinated by CCUH [6,7]. Five orphan drugs (14.7%) were provided within this program in 2010-2014: Elaprase, Cystadane, Increlex, Kuvan and Aldurazyme. However, after reaching the age of 18 and moving into adult patient group, most patients lose state support for reimbursement of medications and rehabilitation services. Another positive example of rare disease management in Latvia is a care of children with cystic fibrosis with a multidisciplinary team approach involving paediatricians, geneticists, pulmonologists, psychologists and social workers.

Orphan medicinal products are partially available via the reimbursement system. Glivec, Sprycel and Tasigna are the only three drugs (8.8%) included in the positive reimbursement list. Glivec was among the first orphan drugs available in Latvia (since 2001). It became first and at that time the only orphan drug included in reimbursement list C and reimbursed for Philadelphia chromosome positive chronic myeloid leukaemia (Ph + CML) second line treatment. Starting from May 2013, Glivec was moved to the reimbursement list A, because cheaper generic drugs became available that changed prescribing and reimbursement criteria for imatinib. Currently it can be prescribed by haematologist or paediatric haemato-oncologist based on the council’s decision and is reimbursed for patients with Ph + CML or acute lymphoblastic leukaemia (Ph + ALL), bone marrow transplant, and gastrointestinal stromal tumours (GIST). Sprycel and Tasigna are reimbursed based on the haematologist council’s decision for adult patients with Ph + CML in the chronic phase if prior therapy with imatinib is not effective (second line treatment).

Not more than two percent of the national drug reimbursement budget is intended to individual reimbursement with limitation up to 14 229 Euro per patient per year. Fifteen orphan medicinal products (44.1%) were provided within this individual reimbursement program in 2008-2012: Arzerra, Atriance, Cystadane, Diacomit, Exjade, Glivec, Mozobil, Nexavar, Nplate, Revatio, Revolade, Sprycel, Sutent, Volibris and Wilzin. Until January 2013, Revolade and Arzerra were reimbursed within the framework of individual reimbursement system. Starting with January 2013, regulation of the Cabinet of Ministers on individual drug reimbursement was changed. According to the new regulation the individual reimbursement can be provided only in cases when diagnosis is not included in the reimbursement list or the diagnosis is included in the list, but there are no drugs included in the reimbursement list for treatment of this diagnosis. In case of Arzerra and Revolade both diagnoses, chronic lymphocytic leukaemia (CLL) and immune (idiopathic) thrombocytopenic purpura (ITP), respectively, are included in the reimbursement list with some therapy alternatives available, making Arzerra and Revolade practically inaccessible for patients. Similar situation may refer to other orphan drugs previously provided within the individual reimbursement mechanism. For instance, only about a half of Volibris price is covered by the state within the individual reimbursement system, while the rest is provided by the company or paid by patients. In case of Nexavar the state coverage is even smaller accounting in less than a quarter of price, therefore it is almost exclusively bought by individual patients for their own money (for some patients a quarter of price is paid by the NHS, but the rest is paid by the manufacturer). However, there are some positive exceptions, for example, reimbursement costs of Revatio do not exceed limit of 14 229 Euro and it is therefore fully covered by the state. Besides, Revatio was reimbursed most frequently among all orphan drugs within the individual reimbursement system in 2008-2012.

A significant problem faced by rare disease patients is associated with special nutrition and medical foods. While some of these foods are vital for rare disease patients, they are usually not classified as medicinal products in Latvia. For some rare diseases medical food often is the main or even the only way in which food is taken, and its discontinuation can be life-threatening. So far there is no standard procedure (regulation) in place for registration of medical food in Latvia [7], and the manufacturer can choose the most appropriate registration procedure to him, most often based on cost considerations. As a result, it is not possible to adapt the same reimbursement arrangement for these foods as it is for medicinal products included in the national reimbursement drug list.

## Discussion

Development of the national plan for rare diseases seems to be an important initial step towards improving situation in this field in Latvia. However, there are currently no official designated centers of expertise as well as no specific register for rare diseases, making it impossible to fully collect and evaluate information on rare diseases. Newborn screening for only two rare disorders is certainly not sufficient resulting in recent discussions concerning expanding of the screening by using tandem mass spectrometry method. The situation appears to be similar in other Baltic States. Lithuanian national plan for rare diseases was approved in 2012 [8] and it was the first plan in Baltic region. Estonian plan was finalized and submitted to national authorities in 2013. Lithuania and Estonia are among those countries with a low neonatal screening coverage. Neonatal screening programs are currently implemented only for phenylketonuria and congenital hypothyroidism. There are also plans to expand screening and introduce tandem mass spectrometry analysis in both countries. There are currently no official designated centers of expertise for rare diseases in all Baltic States. Some centers are recognized by reputation only. Latvia and Lithuania have plans to designate centers of expertise for rare diseases in the future, while Estonia has no such plans. Similarly to Latvia, Lithuania is planning to implement the e-health information system and establish electronic platform based disease registers (including rare diseases). In Estonia, all health related information is already collected in the electronic health information system, which can be updated and used to extract statistical data about rare diseases.

It has been found that even in such small country as Latvia there are some regional differences in availability of diagnostics, treatment and rehabilitation services for some rare diseases, since university hospitals, scientific and research institutions are concentrated in the capital and the largest city of Riga. There are also differences in availability of health care services between paediatric and adult patient groups, giving that five orphan drugs were provided within the CCUH program. Patient organizations play an important role in increasing awareness and providing necessary pressure on society and policy makers. Several patient organizations support patients and represent their interests, including general rare disease patient organization “Caladrius” and patient organizations for specific rare disorders. Public awareness is also supported via Orphanet national website.

Orphan medicines are distributed by both hospital and community pharmacies. Currently 34 orphan drugs are available, including 6 drugs that are no longer considered to be orphan medicines in the EU, concluding that majority of orphan drugs authorized in the EU are not available in Latvia. Orphan medicinal products are partially available via the reimbursement system, with only three drugs included in the positive reimbursement list, all indicated for Ph + CML. Another pathway for getting orphan drugs is individual reimbursement with limitation up to 14 229 Euro per patient per year. Fifteen orphan medicinal products were provided within this program. Annual limit of 14 229 Euro per patient is certainly not sufficient considering high price of orphan drugs. Therefore treatment costs often exceed this limit and the rest of expenses not covered by the NHS should be paid by the patient or provided by the manufacturer. The situation with access to orphan drugs has even worsened with recent changes in terms and conditions of new regulation on the individual reimbursement making some orphan drugs practically inaccessible for patients. Another significant issue is lack of standard procedure (regulation) for registration and reimbursement of special nutrition and medical foods.

Denis et al. compared rare disease and orphan drug markets in six European countries [24]. France, Italy, Sweden and UK have dedicated centers of reference for rare diseases. Besides, there are no official centers of reference in Belgium and the Netherlands, but several medical centers fulfill this role. France, Italy and the Netherlands have implemented additional policy measures and research incentives to promote research and development of orphan medicines. Marketing authorization of orphan drugs is responsibility of the EU, but France has in place a domestic procedure for authorization of orphan products (for temporary use before getting approval from EMA). There are programs for compassionate use of orphan drugs in Belgium, France, Italy, the Netherlands, and UK, but there is no legislation governing compassionate use in Sweden. Some similarities and differences can be found when comparing Latvian situation with the above mentioned European countries. For example, there are no official centers of reference in Latvia, but several medical centers fulfill this role similarly to the process in Belgium and the Netherlands. Generally some funding is available for rare diseases and several research projects take place in Latvia, although these funds and incentives are not specifically designed for rare disease research. As in most EU countries there is no domestic orphan drug authorization procedure, but several programs for orphan drug compassionate use take place in Latvian hospitals. Belgium, France, Italy and the Netherlands compare the price of orphan medicine with the price in other countries [24], while Sweden and UK have free pricing system. All countries included in the above mentioned study consider the budget impact of orphan drugs in the reimbursement application, except Sweden, whereas cost-effectiveness is considered in all countries, except Belgium. In Latvia, there is no specific policy for the pricing and reimbursement of orphan drugs. The NHS evaluates therapeutic value, price, expected budget impact and cost-effectiveness for each drug before it is included in the reimbursement list. Drug price is compared with prices in the following EU countries: Czech Republic, Denmark, Romania, Slovakia, Hungary, Estonia and Lithuania. Comparison with these mostly smaller Eastern European countries (excluding Denmark) seems to be very logical taking into consideration geopolitical location of Latvia, lower GDP level, small population and market size. It can be expected that Latvian situation is likely to be closer to these countries.

Some studies in the field of rare diseases conducted in other Eastern and Southern European countries recently came to light. For instance, in Bulgaria there is no national register or centers of expertise for rare diseases [25]. Regulation of compassionate or off-label use of orphan drugs is also missing. Price of orphan medicines which are going to be included in the reimbursement list is compared with reference prices in a set of EU countries (including Baltic States). Before inclusion in reimbursement list drugs are also assessed for therapeutic value and social significance, but cost-effectiveness of the drug is not considered. The allocation of funds for orphan medicines is made once per year and it is based on the previous year’s budget. Neither national register, nor policy measures, and research incentives for rare diseases exist in Serbia [26]. Cost-effectiveness, budget impact, and the need for a given treatment are taken into account when assessing reimbursement application of a drug. Orphan medicinal products are reimbursed from the national health insurance fund, which reimburses only medicines that are registered in Serbia [8]. There is no centralized marketing authorization procedure in place since Serbia is not the EU Member State. Domestic registration is therefore required, that can take additional time and delay patient access to orphan drugs. Compassionate and off-label use is not recognized by the health insurance system in Serbia, although there is a special fund for reimbursement of the treatment of paediatric patients with metabolic diseases requiring enzyme replacement therapy. Around 2.6 million Euro was planned for this purpose in 2014.

31 orphan drugs were marketed in Belgium in 2008 [24]; 35 drugs in France in 2007; 23 drugs in Italy in 2007; 40 drugs in the Netherlands in 2008; 28 drugs in Sweden in 2008; and 20 drugs in UK in 2006. The situation on orphan drug reimbursement in studied countries was as follows: 32 orphan drugs were reimbursed in Belgium (2009); 35 in France (2007); 21 in Italy (2007); 32 in The Netherlands (2009); 28 in Sweden (2008); and 12 in Scotland (2008). Current study was conducted in 2014 and it found that 34 orphan drugs (28 pure orphan products) are available in Latvia, and only three orphan drugs are included in the positive reimbursement list. This number is extremely small especially considering time difference between the studies and growing number of orphan medicinal products. There were 47 orphan drugs on the European market by the end of 2008 [24], while 72 orphan drugs are currently authorized in the EU.

Surveys on orphan drug availability in Europe had pointed out unacceptable delays and inequalities in rare disease patients’ access to their medicines. Especially countries with a small population suffer from a longer delay in availability of drugs. In 2007, overall lowest availability of orphan drugs was demonstrated in Estonia and Lithuania [27]. In 2010, the number of patients with potential access to orphan drugs ranged from 34% in Greece up to 98% in France [28]. The price also varied between the countries, and in some countries it was up to 160% higher than the lowest European price. Another European study found that differences in annual costs per patient between the EU countries for a given orphan drug may reach 70% [29]. Newer EU Member States are often facing budget restrictions with healthcare budgets much lower than compared to older Member States [30], thereby reimbursement levels can differ. Thus number of available (marketed) orphan drugs in Bulgaria was 22 and 16 of them were accessible (reimbursed) for patients in 2011 [25]. Iskrov et al. point out that this is an important issue especially for Eastern European countries, as a big part of orphan drugs are not priced and reimbursed in many countries. In this geographical and economical region the price level of orphan drugs is not among the lowest in the EU, and that could be explained by the small market size represented by these countries. Serbia might be mentioned as another example, where only four orphan medicines were reimbursed [26]. Authors also suggest that gross domestic product (GDP) value may partly explain differences in the level of orphan drug reimbursement among the European countries, since Serbia is a country with a low GDP. Baltic States are also among the countries with a low GDP. In Lithuania, budget assigned for reimbursement of orphan medicines is limited and insufficient (1.89 million Euro in 2006) [31], therefore access to health care services and orphan drugs in some cases is restricted. 29 orphan medicinal products were marketed in Lithuania in 2011 [8], and about 3 million Euro was allocated for reimbursement of medicinal products and devices for rare diseases in 2013. There is no specific pricing and reimbursement policy for orphan drugs in Estonia. They are reimbursed on the same basis as other medicines. 20 orphan drugs are fully reimbursed by the Estonian health insurance fund. The fund has also reimbursed off-label drugs and medical foods for rare disease patients.

As stated by Drummond et al., because of the small market, orphan drugs are often very expensive [32]. High price may also originate from marketing exclusivity, as well as costs of research and development, that should be recovered from a small number of patients. With standard economic evaluation, these drugs usually do not prove to be cost-effective and it, taking into account their high price, means that patient access may be limited. According to Picavet et al. orphan designated drugs have higher median price (138.56 Euro) than non-designated drugs (16.55 Euro) for rare disease indications [33]. Moreover, price of an orphan drug is higher for a disease with a lower prevalence [29]. Although orphan drugs with an alternative have lower annual cost per patient than those without an alternative [34]. Pharmaceutical companies have to comply with different pricing and reimbursement approaches in each EU country, thereby raising the price of orphan drugs [35]. Moreover, prices of drugs distributed through the hospital pharmacies are not regulated in most European countries, but are negotiated directly between the manufacturer and the hospital [34]. According to Simoens, there is a need for a transparent and evidence based approach towards pricing and reimbursement of orphan drugs.

The economic impact of orphan drugs on national budget is growing, for example, in France representing a total budget of 1 billion Euro in 2009 [30]. The annual per patient cost of orphan drugs varied between 1 251 Euro and 407 631 Euro, with the median cost being 32 242 Euro [36]. The share of the total European pharmaceutical market represented by orphan drugs was 3.3% in 2010, and it was predicted by Schey et al. to increase to a peak of 4.6% in 2016. Another analysis estimated that orphan drugs constituted 1.9% of total drugs expenditure in Belgium in 2008, and predicted it to increase to about 4% in 2013 [37]. While the average budget impact of orphan drugs accounted for 1.7% of the total pharmaceutical expenditure across France, Germany, Italy, Spain and the UK in 2007 [38]. In recent study the observed orphan drug budget impact was 2.5% of total pharmaceutical sales in Sweden and 3.1% in France in 2012 [39]. Precise impact of orphan drugs on Latvian budget has not been determined yet. In the above mentioned studies orphan drug budget impact was around 2-3% of total drugs expenditure depending on market, though the studies were mostly focused on countries with a high GDP. It can be estimated that the budget impact of orphan drugs in Latvia, as a country with a low GDP, is smaller taking into account budgetary restrictions, small market size, and high price of orphan drugs. Recent study evaluated whether a country’s GDP and health technology assessment (HTA) organization influences the orphan drugs’ market uptake [40]. Market volumes were highest in countries with a high GDP. Latvia was included in the cluster of countries with a low GDP and a formal HTA organization together with Hungary and Poland. This cluster had the lowest sales of orphan drugs as well as the lowest contribution of total orphan drug market sales. Other Baltic States were included in the cluster with a low GDP and no HTA organization. For this group of states sales of orphan drugs and contribution of total orphan drug market sales were the second lowest. It should be noted that only five out of 17 orphan drugs selected for the analysis were launched in Latvia, whereas all orphan drugs were launched in France, Spain and the UK. The sales also varied substantially. In Latvia, only 8 000 Euro was spent per 100 000 inhabitants on orphan drugs compared to approximately 560 000 Euro in France. The contribution of orphan drug sales to total market sales was also lowest in Latvia, and percentages varied from 0.07% in Latvia to 1.90% in Estonia. However, the authors acknowledged that this study suffered from several limitations, data restrictions and differences in data quality between the countries. As well not all orphan drugs available in Latvia were included in the analysis. 17 orphan drugs were selected and only five of them were recognized as launched in Latvia. According to data from the current study there are 34 orphan drugs (28 pure orphan products) available on Latvian market. Moreover, Glivec, Elaprase and Revatio were not selected in the above mentioned study. These drugs could have a major impact on total orphan drug market sales in Latvia. Elaprase and Revatio were purchased most frequently among all orphan drugs within the CCUH program and individual reimbursement system, respectively. Glivec was the first and, until recently, the only orphan drug included in the positive reimbursement list. This blockbuster anticancer drug accounted for 34% of total orphan drug sales between 2000 and 2012 in Sweden, and for 27% in France [39]. However, Glivec is no longer considered to be orphan medicine in the EU since 2012, as well generic analogues of imatinib became available in 2013. This fact can affect the price and sales of the product. Considering dynamic nature, and between countries differences in orphan drug markets (and drug markets in general), budget impact of orphan drugs in Latvia should be assessed more accurately. Specificity of the small market with a low GDP, peculiarities of drug reimbursement system, and budgetary restrictions faced by health care system should be taken into account.

## Conclusions

The majority of orphan drugs authorized in the EU are not available in Latvia, moreover those drugs that are available are often not accessible because they are insufficiently reimbursed by the state, and are too expensive to be covered by patients. Diagnostics of rare diseases (including newborn screening) also require improvements. The development and approval of the national plan for rare diseases is an important step towards improving the situation in the field of rare diseases. However, there is still a lot to do and further action is required to improve access to information on rare diseases for both health care professionals and patients. The creation of a rare disease patient register and centers of expertise would allow to collect and evaluate information on specific rare diseases, that would greatly improve the current situation and coordination of further activities in the field. Data collected through the register should also be used to assess the long-term effectiveness and cost-effectiveness of orphan medicines and should be harmonized at the EU level. Early detection and prevention of rare diseases, integrated health care for patients, timely access to orphan drugs and continuing education of health care professionals play crucial role in improving quality of life of patients suffering from orphan diseases. Many of these activities are included in the national plan for rare diseases and most of them are currently in the process of development.

## Abbreviations

CCUH: Children’s Clinical University Hospital
CDPC: Center for Disease Prevention and Control
CLL: Chronic lymphocytic leukaemia
COMP: Committee for orphan medicinal products
CTEPH: Chronic thromboembolic pulmonary hypertension
Dyscerne: European Network of Centers of Expertise for Dysmorphology
EMA: European Medicines Agency
E-Rare: ERA-Net for research programs on rare diseases
EU: European Union
EUCERD: European union committee of experts on rare diseases
EUHASS: European haemophilia safety surveillance
EUROCARE-5: European cancer register based study on survival and care of cancer patients
EUROCAT: European surveillance of congenital anomalies
FISH: Fluorescence in situ hybridization
GDP: Gross domestic product
GIST: Gastrointestinal stromal tumours
IRDiRC: International rare disease research consortium
ITP: Immune (idiopathic) thrombocytopenic purpura
LBRSC: Latvian Biomedical Research and Study Center
MAH: Marketing authorization holder
MEN: Multiple endocrine neoplasia
MODY: Maturity onset diabetes of the young
MoH: Ministry of health
MPS: Mucopolysaccharidosis
NHS: National health service
PAAIR: Patient associations and alpha-1 international register
PAH: Pulmonary arterial hypertension
Ph + ALL: Philadelphia chromosome positive acute lymphoblastic leukaemia
Ph + CML: Philadelphia chromosome positive chronic myeloid leukaemia
pNET: Neuroendocrine tumours of pancreatic origin
PSCUH: Pauls stradins clinical university hospital
RARECARENet: Information network on rare cancers
RCC: Renal cell carcinoma
RECUH: Riga East Clinical University Hospital
RSU: Riga Stradins University
SAM: State agency of medicines
